# Origin of Human T-Lymphotropic Virus Type 1 in Rural Côte d’Ivoire

**DOI:** 10.3201/eid1805.111663

**Published:** 2012-05

**Authors:** Sébastien Calvignac-Spencer, Edgard V. Adjogoua, Chantal Akoua-Koffi, Claudia Hedemann, Grit Schubert, Heinz Ellerbrok, Siv Aina Jensen Leendertz, Georg Pauli, Fabian H. Leendertz

**Affiliations:** Robert Koch-Institut, Berlin, Germany (S. Calvignac-Spencer, C. Hedemann, G. Schubert, H, Ellerbrok, S.A. Jensen Leendertz, G. Pauli, F. H. Leendertz);; Institut Pasteur, Abidjan, Côte d’Ivoire (E.V. Adjogoua, C. Akoua-Koffi)

**Keywords:** human T-lymphotropic virus type 1, HTLV-1, simian T-lymphotropic virus type 1, STLV-1, zoonoses, nonhuman primates, western Africa, Taï National Park, Côte d’Ivoire, viruses

## Abstract

Simian T-lymphotropic virus type 1 (STLV-1) strains occasionally infect humans. However, the frequency of such infections is unknown. We show that direct transmission of STLV-1 from nonhuman primates to humans may be responsible for a substantial proportion of human T-lymphotropic virus type 1 infections in rural Côte d’Ivoire, where primate hunting is common.

Human T-lymphotropic virus type 1 (HTLV-1) can induce adult T-cell leukemia or lymphoma and HTLV-1–associated myelopathy or tropical spastic paraparesis. These pathologies are a serious threat to the several million persons infected with HTLV-1 (*1*).

Although HTLV-1 has spread globally, its geographic distribution is not uniform. Most infected persons live in areas where the virus is endemic and seroprevalence is comparatively high (>1%) (*1*), i.e., in Japan, Melanesia, South America, the Caribbean, and sub-Saharan Africa. Phylogenetic analyses demonstrate that the geographic distribution of HTLV-1 genetic diversity is also not uniform. The most genetic diversity is seen in sub-Saharan Africa, where 6 of the 7 human molecular subtypes (HTLV-1A, B, D, E, F, and G) are found. Of those 6 subtypes, 5 are mainly found in or endemic to central Africa: HTLV-1B, D, E, F, and G (*1*).

Molecular HTLV-1 subtypes from humans in central Africa belong to composite clades that comprise HTLV-1 strains and simian T-lymphotropic virus type 1 (STLV-1) strains derived from nonhuman primates (*2*). Nonhuman primates in Africa are considered to be the source of recurrent zoonotic transmissions of STLV-1 to local human populations; virus transmission is believed to occur during the collection and consumption of nonhuman primate bushmeat. This belief is supported by the fact that self-reported nonhuman primate hunters in Cameroon were infected with viruses closely related to STLV-1 strains circulating among local nonhuman primate prey (*3*). However, because intrafamilial transmission of HTLV-1B and -1D was also documented among hunters-gatherers in Cameroon (*4*), it is impossible to sort out cases of direct zoonotic transmission of STLV-1 from cases of consecutive human-to-human spread of virus (evolutionary rates for HTLV-1/STLV-1 are very slow) (*5*).

However, HTLV-1 and STLV-1 strains from western African segregate clearly in phylogenetic analyses; most humans are infected with HTLV-1A, the only human-restricted molecular subtype (*6**–**9*). Therefore, in western compared with central Africa, human infections with viruses closely related to local STLV-1 strains are much more likely to reflect direct zoonotic transmission. This situation enabled us to investigate the frequency of such direct zoonotic transmissions in a rural region of Côte d’Ivoire neighboring the Taï National Park (Figure 1).

**Figure 1:**
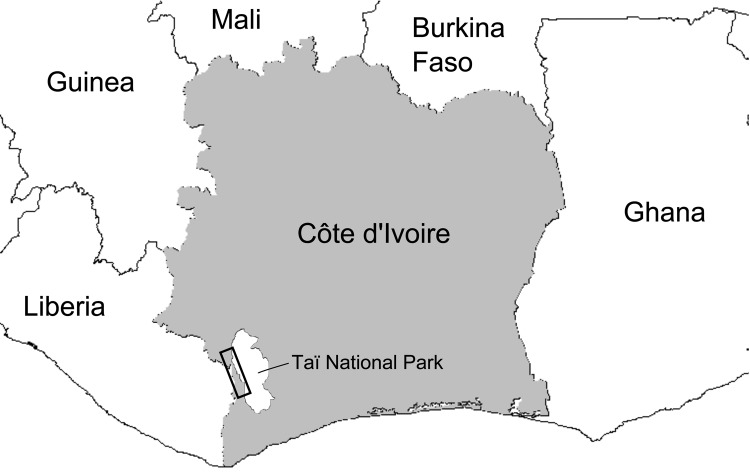
Sampling zone in study of the origin of human T-lymphotropic virus type 1 in rural western Africa, 2006–2007. Taï National Park is indicated in white on the gray background of Côte d’Ivoire. The black rectangle overlapping Taï National Park defines a zone encompassing the 18 villages where study participants resided. Village names and the number of participants are as follows: Daobly (38), Djereoula (31), Djiboulay (40), Gahably (55), Gouléako (37), Goulégui-Béoué (55), Kéibly (90), Pauléoula (20), Ponan (21), Port-Gentil (47), Sakré (75), Sioblooula (35), Taï (26), Tieleoula (40), Zagné (17), Zaïpobly (37), Ziriglo (74).

## The Study

During 2006–2007, blood samples were obtained from 776 volunteers living in 18 villages bordering Taï National Park. All participants signed informed consent forms and completed questionnaires aimed at determining their exposure to nonhuman primate bushmeat through activities such as hunting of nonhuman primates or consumption of nonhuman primate bushmeat.

To determine effective exposure to HTLV-1/STLV-1, we used an HTLV-1/2 ELISA to test serum samples for reactivity to HTLV-1/2 antigens (*10*). Of the 776 serum samples, 16 were positive according to the ELISA manufacturer’s criteria; an additional 15 samples had values just below the cutoff. We extracted DNA from all ELISA-reactive samples and performed a search for HTLV-1/STLV-1 sequences by using a *tax*-specific quantitative PCR (*8*). Of the 31 samples, 10 were positive and were analyzed by using a multiplex nested/seminested PCR targeting *env* and long terminal repeat (LTR) sequences (Table 1; Technical Appendix). To identify multiple infections with HTLV-1/STLV-1, this assay was applied on near endpoint dilutions of the 10 DNA extracts (2–6 starting template molecules per reaction; Technical Appendix). For each person, 6–20 *env* and 2–20 LTR sequences (15–40 sequences per person) were determined by Sanger sequencing. No evidence of multiple infections was found.

**Table 1:** Characteristics of persons positive for HTLV-1 or STLV-1 in a study of the origin of HTLV-1, rural western Africa, 2006–2007*

Study participant†, sex	Infecting subtype	Minimum observed distance to any STLV-1, %						
					Type of contact and nonhuman primate contacted			
		LTR	*env*		Hunting	Dismembering	Preparation or cooking	Eating
Gah050, M	STLV-1I/SM	0.6	0.4		None	Monkeys, chimp	Monkeys, chimp	Monkeys, chimp
Gul014, F	HTLV-1A	3.2	2.9		None	None	Monkey, chimp	Monkeys, chimp
Kei005, F	STLV-1I/SM	0.6	0.5		None	Monkeys	Monkeys	Monkeys
Kei025, M	STLV-1J	0.3	0.2		None	Monkeys	None	Monkeys
Kei075, F	HTLV-1A	3.0	3.0		None	None	Monkeys	Monkeys
Pau002, F	HTLV-1A	3.2	2.5		None	None	Monkeys	Monkeys
Pau009, M	STLV-1I/SM	0	0.2		Monkeys, chimp	Monkeys, chimp	None	Monkeys, chimp
Pon002, F	HTLV-1A	4.2	2.8		None	Monkeys	Monkeys	Monkeys
Tie005, F	HTLV-1A	4.0	2.5		None	Monkeys	Monkeys	Monkeys
Tie011, F	HTLV-1A	4.5	2.4		None	Monkeys	Monkeys	Monkeys

*Gray shading indicates infections with STLV-1–like HTLV-1 (as determined through phylogenetic analyses). Minimum distances were calculated by using the same datasets as for phylogenetic analyses (see Technical Appendix). HTLV-1, human T-lymphotropic virus type1; STLV-1, simian T-lymphotropic virus type1; LTR, long terminal repeat; chimp, chimpanzee(s). †First 3 letters refer to the persons’ village of residence.

**Figure 2:**
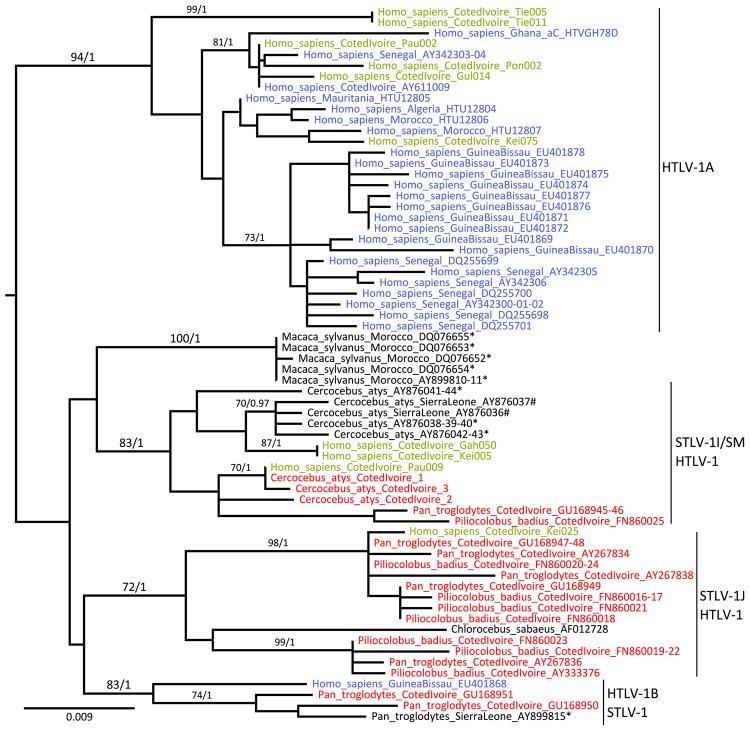
Maximum-likelihood tree based on the analysis of a long terminal repeat (853 bp) alignment in a study of the origin of human T-lymphotropic virus type 1 (HTLV-1) in rural western Africa, 2006–2007. Bayesian analyses supported similar topologies. After rooting, branches leading to outgroup sequences (HTVMEL5 and Z46900) were removed from the figure to increase its legibility. The HTLV-1 sequences determined from specimens from West and North Africa are shown in light blue; HTLV-1 sequences determined from persons living in the region of Taï National Park are shown in green; STLV-1 sequences determined from specimens from West and North Africa are shown in black; and STLV-1 sequences determined from persons living in the Taï National Park are shown in red. Sequence names are built as follows: [host species]_[country of origin]_[GenBank accession number]. Reference sequence names also include their molecular subtype assignation: [host species]_[country of origin]_[molecular subtype]_[accession number]. *Sequences determined from captive or semicaptive hosts; #sequences determined from bushmeat samples. Molecular subtypes were assigned on the basis of an analysis performed on an enlarged dataset including assigned reference sequences (data not shown). Bootstrap (Bp) and posterior probability (pp) values are indicated where Bp>50.0 and pp>0.95. Scale bar indicates nucleotide substitutions per site.

Phylogenetic analyses were performed by using Bayesian and maximum likelihood methods on *env* and LTR datasets (Technical Appendix). Both methods agreed on all essential features of the LTR tree topology (Figure 2) and *env* tree topology (Technical Appendix Figure). Six of the newly determined HTLV-1 sequences were unambiguously related to HTLV-1A (bootstrap, 94; posterior probabilities, 1) (Figure 2), confirming the predominance of this molecular subtype in Côte d’Ivoire and in western Africa (*6*). Another 3 HTLV-1 sequences were closely related to STLV-1 sequences found in sooty mangabeys (*Cercocebus atys*) from Taï National Park (bootstrap, 83; posterior probabilities, 1) (Figure 2; Technical Appendix Table 2), whereas the last 1 was related to STLV-1 sequences from red colobus monkeys (*Piliocolobus badius badius*) and chimpanzees (*Pan troglodytes verus*) from Taï National Park (bootstrap, 98; posterior probabilities, 1) (Figure 2; Technical Appendix Table 2) (*7**,**8*). Bayesian analyses were run under the assumption of a molecular clock and calibrated. However, reliable divergence dates could not been determined because most shallow nodes of the trees, including those of interest here, were not supported (Technical Appendix). Observed divergences, however, seemed compatible with cross-species transmission events, particularly in the case of study participant Pau009 (divergence to closest STLV-1, 0% in LTR and 0.2% in *env*) (Table 1).

## Conclusions

We investigated the frequency of direct zoonotic transmission of STLV-1 in a rural region of Côte d’Ivoire neighboring Taï National Park and found that only 2 of the STLV-1–related sequences would be compatible with a local human-to-human transmission (Gah050 and Kei005; Figure 2). Therefore, our data support the notion that direct zoonotic transmissions of STLV-1 represent a measurable proportion of HTLV-1 infections, at least in rural regions bordering nonhuman primate habitat. In addition, these results mirror observations made among adult chimpanzees from Taï National Park, which are often infected with retroviruses (i.e., simian foamy viruses and STLV-1) of their prey (Figure 2) (*7**,**11*).

Despite the high prevalence of STLV-1, simian foamy virus, and simian immunodeficiency virus infections among red colobus populations (*8*) and the fact that this nonhuman primate species is the one most frequently hunted by humans (*4*), most zoonotic transmissions of retroviruses in western Africa seem to originate from sooty mangabeys, as shown here for STLV-1 and previously described for simian immunodeficiency virus of sooty mangabeys, the precursor of HIV-2 (*12*). It remains to be determined whether these zoonotic transmissions from sooty mangabeys are favored as a result of molecular determinants (e.g., convergent evolution of retroviral receptors) or behavioral determinants (e.g., increased aggressiveness).

Considering the human exposure to nonhuman primate bushmeat in this region (as illustrated by ≈150,000 kg sold per year in markets) (*13*) and given the high prevalence of STLV-1 among local nonhuman primates (*7**,**8*), the observation that zoonotic transmission events are, in absolute terms, exceedingly rare is striking. Yet, the accumulation of genetically distinct HTLV-1/STLV-1 over restricted geographic areas remains possible, as illustrated by the finding of 1 person infected with HTLV-1A and 2 persons infected with putative STLV-1 in a single village, Kéibly (Table 1; Figure 1). Such local accumulations add to the threat represented by direct transmissions of STLV-1 because they can provide an opportunity for recombinant viruses to emerge, even though HTLV-1/STLV-1 biology may be unfavorable to recombination (*14*).

The analysis of behavioral data reveals generalized exposure of local populations to cooked nonhuman primate bushmeat (Table 2). Exposure to fresh tissues, which can be expected to be more risky in terms of retroviral transmission, is less common (Table 2). Along a gradient of bushmeat freshness, going from hunting to preparation and cooking, a clear reversal of sex-related skew can be observed: only men are hunters, men and women are equally involved in dismembering, and women predominantly prepare and cook nonhuman primate bushmeat (Table 2). Hence, men likely constitute a population at risk. In our study, 75% of persons who were identified as infected with viruses closely related to STLV-1 were men, whereas all HTLV-1A–infected persons were women. Increased surveillance for zoonotic transmission of STLV-1 to humans in areas where such transmission is more likely and increased surveillance of nonhuman primate species with high transmission potential (like sooty mangabeys) will contribute to a better understanding of risk factors.

**Table 2:** Type of nonhuman primate contact by participants in a study of the origin of HTLV-1 in rural western Africa, 2006–2007*

Variable	Activity resulting in contact			
	Hunting	Dismembering	Preparing or cooking	Eating
Women, n = 402	0%	62.4%	66.9%	81.3%
Men, n = 371	11.6%	63.6%	21.6%	90.8%
Relative exposure, men vs. women	NA	1.02	0.32	1.12

*Sex assignation was lost for 3 persons; thus, the total sampling size was 773 rather than 776, the total number included in the study. HTLV-1, human T-lymphotropic virus type1; NA, not applicable.

## Supplementary Material

Technical Appendix Detailed material and methods for a study on the origin of human T-lymphotropic virus type 1 in rural western Africa, 2006–2007, and a table showing Simian T-lymphotropic virus type 1 infection in nonhuman primates from Taï National Park, Côte d'Ivoire.
