# Supplementary material for: Origin of Human T-Lymphotropic Virus Type 1 in Rural Côte d’Ivoire
Source: Emerg Infect Dis. 2012 May;18(5):830–3. doi: 10.3201/eid1805.111663 (PMC3358045; doi:10.3201/eid1805.111663)
Supplement: Technical Appendix — Detailed material and methods for a study on the origin of human T-lymphotropic virus type 1 in rural western Africa, 2006–2007, and a table showing Simian T-lymphotropic virus type 1 infection in nonhuman primates from Taï National Park, Côte d'Ivoire. [file 11-1663-Techapp_8p.pdf]

Article DOI: http://dx.doi.org/10.3201/eid1805.111663

Origin of Human T-Lymphotropic Virus Type 1 in Rural Côte d'Ivoire

Technical Appendix

Detailed material and methods for a study on the origin of human T-lymphotropic virus type 1 in rural West Africa

Sample Collection and Storage

Nonhuman primate samples were collected as described in (*1*). People living in 18 villages bordering the Taï National Park, Côte d'Ivoire, were invited to participate in this study. Potential participants received thorough, coherent information about the study. After having given their informed consent, study participants were asked to complete a questionnaire that attempted to determine each individual's involvement in various steps of nonhuman primate bushmeat processing, such as hunting of nonhuman primate or consumption of nonhuman primate bushmeat. Blood was then collected by venipuncture from participants before being separated into plasma and cellular fractions. Buffy coat layer and plasma were isolated and immediately snap-frozen in liquid nitrogen. At the end of the field study, buffy coat and plasma samples were transferred to Institut Pasteur (Abidjan, Côte d'Ivoire) and Robert Koch-Institut (Berlin, Germany) and kept at –80°C in each of the institutes until analyses were performed.

Ethics

Permits for nonhuman primate sampling were obtained from the Office Ivoirien des Parcs et Réserves and the Ministère de la Recherche of Côte d'Ivoire. Permission for the study was obtained from the Institut Pasteur (Abidjan, Côte d'Ivoire) and the Ministère de la Santé of Côte d'Ivoire, representing the ethics commission (Ref #: 0428/MDCS/CAB-1/kss). The study was performed according to the Declaration of Helsinki, "Ethical Principles for Medical Research Involving Human Subjects," as last revised by the World Medical Association. Informed consent

forms were signed by all participants after the scope of the study was explained in the local language.

Serology

Serum samples were tested in duplicate for HTLV-1 reactivity by using an HTLV-1/2 ELISA (Murex Biotech, Dartford, UK) (*2*) at Robert Koch-Institute, Germany, and in parallel at the Institute Pasteur Côte d'Ivoire.

Molecular Biology

DNA was extracted from blood-isolated buffy coat by using a DNA blood kit and from the mangabey samples by using a DNeasy tissue kit (QIAGEN, Hilden, Germany). First, 200 ng DNA extract was used as template in a quantitative PCR targeting a short *tax* fragment (≈190 bp), by using the primers SK43 (CGG ATA CCC AGT CTA CGT GT) and SK44 (GAG CCG ATA ACG CGT CCA TCG) and the probe HTLV TaxTM (6FAM-CGC CCT ATG GCC ACC TGT CCA GA XT P; 6FAM is 6-carboxyfluorescein), following the protocol described in (*1*).

Next, longer long terminal repeat (LTR) and *env* proviral DNA fragments (≈650 bp and 560 bp, respectively) were amplified by using a multiplex seminested/nested PCR system to determine phylogenetically informative sequences. In first-round reactions, two pairs of primers were used: S10 (GGC CCT AAT AAT TCT ACC CG) and R8906 (GAA CTT TCG ATC TGT AAC GGC G) for LTR; HFL71 (CCA GTG GAT CCC GTG GAG A) and HFL72 (AGG AGG ATT TGA TGG GAG A) for *env*. Cycling conditions were: 95°C for 5 min followed by 35 cycles of 95°C for 30 s, 55°C for 30 s and 72°C for 90 s with a final step at 72°C for 10 min. For human samples, the quantities of DNA extract that were added to this first-round reaction were derived from the results of the HTLV-*tax* quantitative PCR and arranged so that each reaction would start from only 2 to 6 templates. A total of 47 such near endpoint dilution reactions were run for each of the 10 DNA extracts. Our reason for conducting these steps in this manner was that in these conditions, multiple infections would easily be identified by multiple peaks in chromatograms at polymorphic positions. For nonhuman primate samples, first round reactions were seeded with 200 ng DNA extract.

Two separate second-round reactions were then completed by using the first-round products. Primers Xho (GAG CTC GAG CAG ATG ACA ATG ACC ATG AG) and R8906 were used in a seminested reaction that targeted LTR while primers HFL75 (TCA AGC TAT AGT CTC CTC CCC CTG) and ENV2 (GGG AGG TGT CGT AGC TGA CGG AGG) were used in a nested reaction that targeted *env*. Cycling conditions were 95°C for 5 min followed by 35 cycles of 95°C for 30 s, 58°C (LTR) or 62°C (*env*) for 30 s and 72°C for 90 s with a final step at 72°C for 10 min. Two µL from a 40-fold dilution of first-round reaction products were used to seed all second-round reactions.

PCR products were visualized with gel electrophoresis before being purified. Sequencing was performed according to the Sanger method. For all cases, comparison to publicly available sequences, using the NCBI BLAST service (*3*), confirmed that the expected proviral DNA sequences had been amplified. Sequences determined in this study have been deposited in the EMBL Nucleotide Sequence Database under the following accession numbers: HE667747-59 (LTR) and HE667760-72 (*env*).

Sequence Analyses

Datasets

The LTR and *env* datasets comprised the sequences determined in this study, 2 outgroup sequences (1 STLV-1 sequence determined from an Asian macaque and 1 HTLV-1C determined from a Solomon Islands inhabitant), as well as all publicly available HTLV-1/STLV-1 sequences determined from humans or nonhuman primates in West and North Africa. All publicly available sequences were retrieved from NCBI (in phylogenetic trees, all sequences appear with their accession numbers). Sequences determined from captive monkeys from uncertain geographic origin, such as captive nonhuman primates, were also included as long as the distribution of the host species in the wild was clearly restricted to West and North Africa (according to distribution ranges from the IUCN Red List Web site: http://www.iucnredlist.org/). Table 1 summarizes the main characteristics of the West and North African sequences that were part of these datasets.

Table 1. Main characteristics of publicly available HTLV-1 and STLV-1 sequences determined from West and North African humans and nonhuman primates*

Total cases	HTLV-1, country (no. cases)		STLV-1, host species, country (no. cases)	
	LTR	*env*	LTR	*env*
	Algeria (1), Côte d'Ivoire (1), Ghana (1), Guinea Bissau (11), Morocco (2), Mauritania (1), Senegal (11)	Burkina Faso (3), Côte d'Ivoire (5), Guinea Bissau (2), Mali (1), Mauritania (3), Senegal (20)	*Cercocebus atys*, captive (7), *C. atys*, Sierra Leone (2), *Chlorocebus sabaeus*, captive (1), *Macaca sylvanus*, Morocco (6), *Pan troglodytes*, Côte d'Ivoire (10), *P. troglodytes*, Sierra Leone (1), *Piliocolobus badius*, Côte d'Ivoire (11)	*C. atys*, captive (17), *C. atys*, Sierra Leone (3), *C. sabaeus*, Senegal (4), *M. sylvanus*, Morocco (5), *P. troglodytes*, Côte d'Ivoire (9), *P. troglodytes*, captive (4), *P. badius*, Côte d'Ivoire (1)
Total	28	34	38	43

*HTLV-1, human T-lymphotropic virus type 1; STLV, simian T-lymphotropic virus type 1; LTR, long terminal repeat.

Results of analyses performed on nonhuman primates from Taï National Park are specifically reported in Table 2.

Table 2. Simian T-lymphotropic virus type 1infection in nonhuman primates from Taï National Park, Côte d'Ivoire

Nonhuman primate	No. animals positive/no. total	Reference
Cercocebus atys, sooty mangabey	3/5	This study
Piliocolobus badius badius, red colobus monkey	13/27	(1,2)
Pan troglodytes verus, Western common chimpanzee	11/24	(2,14,15)

Molecular subtype assignation was based on the analysis of an enlarged LTR dataset, which also comprised the 42 reference sequences described in (*4*) (data not shown). Each dataset was aligned by using MUSCLE (*5*) as implemented in SeaView v4 (*6*), reduced to unique sequences by using Fabox (http://birc.au.dk/software/fabox/) (*7*) and checked for the presence of recombinant sequences by using RDP3 (no recombinant was found) (*8*). It should be noted that although alignments included gaps, most were unambiguous. In addition, analytical methods used thereafter are notoriously insensitive to gaps (as long as they do not lead to faulty site homology), which are dealt with as missing data. Haplotyped datasets are available at http://sebastiencalvignac.fr/downloads/index.html.

Phylogenetic Analyses (including Divergence Date Estimations)

Several models of nucleotide substitution were first assessed for their ability to explain the data by using jModeltest v0.1 (*9*). On the basis of the comparison of Akaike information criterion (AIC) scores derived from model likelihoods, a global time reversible (GTR) model with a proportion of invariant sites (+I) and γ-distributed rate heterogeneity with 4 classes (+G4) was

selected for both the LTR and the *env* datasets, Phylogenetic analyses were performed in both maximum-likelihood (ML) and Bayesian frameworks, using the corresponding models.

ML analyses were performed on the PhyML webserver (http://www.atgc-montpellier.fr/phyml/) (*10,11*). Substitution models also included nucleotide equilibrium frequency optimization. Tree search was arranged to start from a BioNJ tree and to be performed using both nearest-neighbor interchange and subtree pruning-regrafting with optimization of topology and branch lengths. Branch robustness was assessed by using non-parametric bootstrapping (500 pseudo-replicates).

Bayesian analyses were performed by using BEAST v1.6.1 (*12*). Analyses were run under the assumptions of a relaxed molecular clock (uncorrelated lognormal) and a constant population size (previous analyses of a comparable HTLV-1/STLV-1 dataset had shown that tree topology was robust to tree shape assumptions) (*1*). So as to be able to place divergence events into an absolute time framework, we placed a strong prior on the divergence date of Melanesian (HTVMEL5) and West African HTLV-1 and STLV-1 (all other sequences except Z46900). The divergence of Melanesian/Australian HTLV-1 strains from all other HTLV-1 strains is indeed thought to reflect a host virus co-divergence event which took place 40,000 to 60,000 years ago when human populations started migrating in direction of the Pacific islands (*13*). We therefore chose to model the time to the most recent common ancestor of all HTLV-1 strains by using a normal distribution of mean 50,000 years and SD 5,100 years, which resulted in a distribution for which 95% of the values were to be included between 40,000 years and 60,000 years. Three runs totaling 40,000,000 generations were completed for the LTR dataset and 2 runs totaling 200,000,000 generations were completed for the *env* dataset. Trees, and numerical values taken by parameters of the model were sampled every 1,000 generations. Tracer v1.5 (http://tree.bio.ed.ac.uk/software/tracer/) was used to check that individual runs had converged, that independent runs converged onto the same parameter values and that chain mixing behavior was appropriate (effective sample size values of combined runs >200). Trees sampled in duplicate runs were then gathered into a single file by using LogCombiner v1.6.1 (part of the BEAST suite) after the removal of a visually conservative 10% burn-in period and, a 2- and 10-fold decrease in sampling frequencies for LTR and *env* chains, respectively. The information of 18,000 trees per dataset was condensed into a maximum clade credibility tree by using

TreeAnnotator v1.6.1 (also part of the BEAST suite). Posterior probability, the frequency of a given bipartition in the posterior sample, was taken as a measure of branch robustness.

Tree Display

ML trees were chosen to be displayed as Figure 2 (LTR; main text) and Technical Appendix Figure (*env*; this file). Outgroup-based rooting was further optimized by using Path-O-Gen (http://tree.bio.ed.ac.uk/software/pathogen/), a purely cosmetic operation whose principle is equivalent to midpoint rooting, although being much cleaner since it determines the display that minimizes the variance of root-to-tip distances (as midpoint rooting it therefore makes the assumption of clock-like evolution). Branch robustness measures of ML and Bayesian analyses were annotated on the resulting tree.

References

1. Leendertz SAJ, Junglen S, Hedemann C, Goffe A, Calvignac S, Boesch C, et al. High prevalence, coinfection rate, and genetic diversity of retroviruses in wild red colobus monkeys (*Piliocolobus badius badius*) in Taï National Park, Côte d'Ivoire. J Virol. 2010;84:7427–36. PubMed http://dx.doi.org/10.1128/JVI.00697-10

2. Leendertz FH, Boesch C, Ellerbrok H, Rietschel W, Couacy-Hymann E, Pauli G. Non-invasive testing reveals a high prevalence of simian T-lymphotropic virus type 1 antibodies in wild adult chimpanzees of the Taï National Park, Côte d'Ivoire. J Gen Virol. 2004;85:3305–12. PubMed http://dx.doi.org/10.1099/vir.0.80052-0

3. Altschul SF, Gish W, Miller W, Myers EW, Lipman DJ. Basic local alignment search tool. J Mol Biol. 1990;215:403–10. PubMed

4. Alcantara LC, Cassol S, Libin P, Deforche K, Pybus OG, Van Ranst M, et al. A standardized framework for accurate, high-throughput genotyping of recombinant and non-recombinant viral sequences. Nucleic Acids Res. 2009;37:W634–42. PubMed http://dx.doi.org/10.1093/nar/gkp455

5. Edgar RC. MUSCLE: multiple sequence alignment with high accuracy and high throughput. Nucleic Acids Res. 2004;32:1792–7. PubMed http://dx.doi.org/10.1093/nar/gkh340

6. Gouy M, Guindon S, Gascuel O. SeaView version 4: A multiplatform graphical user interface for sequence alignment and phylogenetic tree building. Mol Biol Evol. 2010;27:221–4. PubMed http://dx.doi.org/10.1093/molbev/msp259

7. Villesen P. FaBox: an online toolbox for FASTA sequences. Mol Ecol Notes. 2007;7:965–8. http://dx.doi.org/10.1111/j.1471-8286.2007.01821.x

8. Martin DP, Lemey P, Lott M, Moulton V, Posada D, Lefeuvre P. RDP3: a flexible and fast computer program for analyzing recombination. Bioinformatics. 2010;26:2462–3. PubMed http://dx.doi.org/10.1093/bioinformatics/btq467

9. Posada D. jModelTest: phylogenetic model averaging. Mol Biol Evol. 2008;25:1253–6. PubMed http://dx.doi.org/10.1093/molbev/msn083

10. Guindon S, Dufayard JF, Lefort V, Anisimova M, Hordijk W, Gascuel O. New algorithms and methods to estimate maximum-likelihood phylogenies: assessing the performance of PhyML 3.0. Syst Biol. 2010;59:307–21. PubMed http://dx.doi.org/10.1093/sysbio/syq010

11. Guindon S, Lethiec F, Duroux P, Gascuel O. PHYML Online–a web server for fast maximum likelihood-based phylogenetic inference. Nucleic Acids Res. 2005;33:W557–9. PubMed http://dx.doi.org/10.1093/nar/gki352

12. Drummond AJ, Rambaut A. BEAST: Bayesian evolutionary analysis by sampling trees. BMC Evol Biol. 2007;7:214. PubMed http://dx.doi.org/10.1186/1471-2148-7-214

13. Lemey P, Pybus OG, Van Dooren S, Vandamme AM. A Bayesian statistical analysis of human T-cell lymphotropic virus evolutionary rates. Infect Genet Evol. 2005;5:291–8. PubMed http://dx.doi.org/10.1016/j.meegid.2004.04.005

14. Junglen S, Hedemann C, Ellerbrok H, Pauli G, Boesch C, Leendertz FH. Diversity of STLV-1 strains in wild chimpanzees (*Pan troglodytes verus*) from Côte d’Ivoire. Virus Res. 2010;150:143–7. PubMed http://dx.doi.org/10.1016/j.virusres.2010.02.020

15. Leendertz FH, Junglen S, Boesch C, Formenty P, Couacy-Hymann E, Courgnaud V, et al. High variety of different simian T-cell leukemia virus type 1 strains in chimpanzees (Pan troglodytes verus) of the Taï National Park, Côte d'Ivoire. J Virol. 2004;78:4352–6. PubMed http://dx.doi.org/10.1128/JVI.78.8.4352-4356.2004

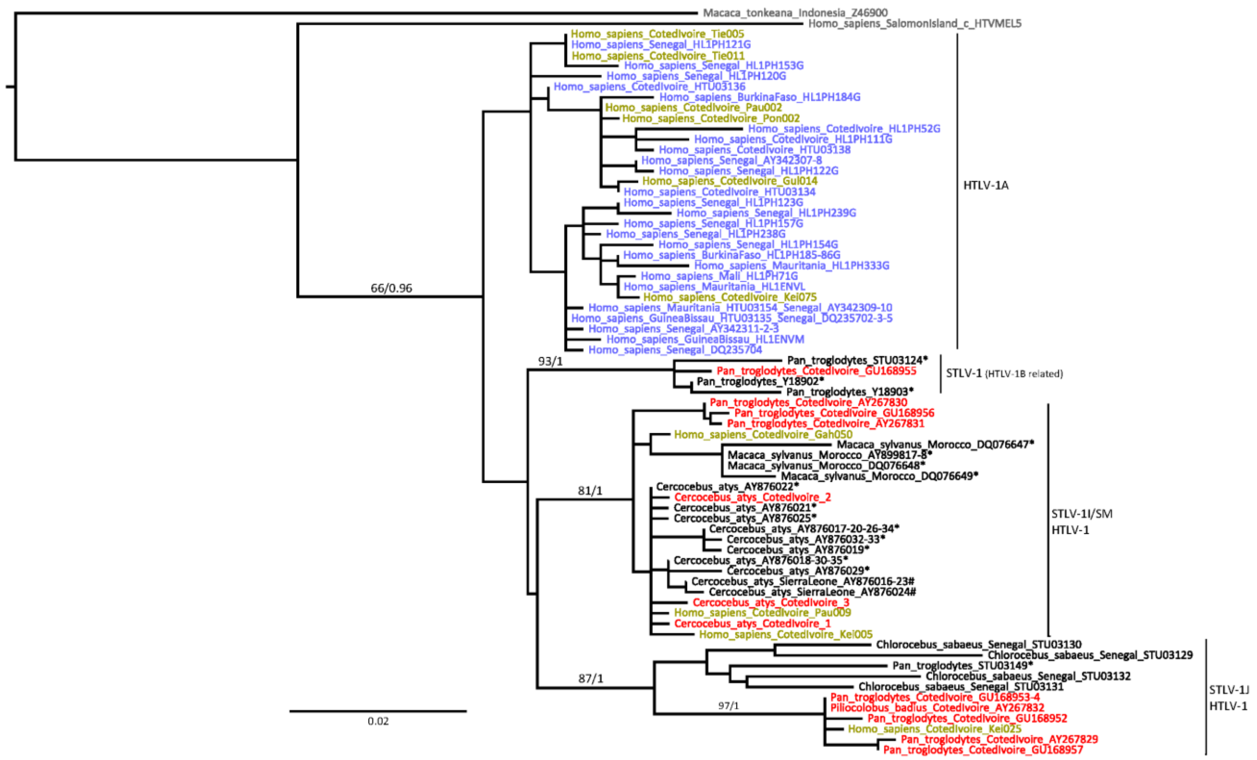

Figure. Maximum-likelihood tree based on the analysis of a 522-bp long *env* alignment, including all available human T-lymphotropic virus type 1 (HTLV-1) and simian T-lymphotropic virus type 1 (STLV-1) sequences from West Africa. Bayesian analyses supported similar topologies. HTLV-1 sequences determined from specimens from persons living in the Taï region are green; HTLV-1 sequences determined from other specimens from West and North Africa are blue; STLV-1 sequences determined from nonhuman primates living in the Taï National Park are red; STLV-1 sequences determined from other specimens from West and North Africa are black. Sequence names are built as in the Figure in the main text. *, sequences determined from captive or semicaptive hosts; #, sequences determined from bushmeat samples. Bootstrap (Bp) and posterior probability (pp) values are indicated where Bp$\geq$50.0 and pp$\geq$0.95.
